# Listening through the learning conversation: a thought provoking intervention

**DOI:** 10.15694/mep.2018.0000199.1

**Published:** 2018-09-06

**Authors:** Mike Davis, Kate Denning

**Affiliations:** 1 Advanced Life Support Group

**Keywords:** Educational strategies, Educational theory, Students/trainees, Active listening, Feedback

## Abstract

This article was migrated. The article was marked as recommended.

Feedback plays an essential role in the learning that occurs on life support courses. Since 2007 the preferred method of managing feedback has been the learning conversation, but it remains an area that many facilitators profess to finding challenging. In this article we will explore how simple conversational techniques involved in active listening can lead to significant learning.

## The importance of feedback

Feedback is a natural feature of the way in which newcomers are introduced to any community they enter and has always been part of the learning process. It is located in a number of classical theories of adult learning, including the notion of learning from experience ( Dewey, 1933, Kolb, 1984) and the contribution of reflection to that process.

In medical education, there has been a move from “stupid boy” (Doctor in the House) to more systematic explorations of performance, both positive and negative ( Pendleton *et al*. 1984), always with the intention of correction of error and seeking improvement. Both of these early models allowed the experienced practitioner to identify and articulate error and the learner to listen and act (or not) on the information in future performance, depending on what is now described as their level of “feedback literacy” (Boud and Molloy, 2015).

What these models have in common is the belief that one person (usually the older and more experienced) has privileged insight into actions and this validates any perspective that they hold. The learning conversation is a challenge to this notion and the basis of the conversation is an acknowledgement that the learner’s perspective should be given greater status if there is to be a more robust basis for future behaviour. The driver, therefore, is not a notional target performance against which a learner’s actions can be judged, but rather a systematic, but personal account that represents the basis of a dialogue designed to analyse and critique performance.

## Devising the learning conversation

The impetus for the learning conversation came about as a response to discomfort on the part of many facilitators to what had unintentionally evolved into a rigid and formulaic process, for example, “Tell me three good things ..”. In contrast, a learning conversation implies that it is the learner, not the facilitator who initially drives the content and the process of feedback and this will often be a spontaneous reaction to the experience. While the facilitator, more expert and with an awareness of learning outcomes in mind, may have an agenda, the expectation is that this is not allowed to dominate. The basis of a productive conversation is one in which the learner takes the lead and it has a natural, unforced flow, typical of any conversation.

Along with much of medical education, feedback has generated a number of mnemonics or acronyms to offer a model that facilitators could follow. For a variety of reasons beyond the scope of this paper, we are a little resistant to these for feedback purposes, seeing them as, at best, problematic structures that prevent all parties engaging with their dominant task, i.e. interacting meaningfully about their recent experience. These devices can be useful memory aides but they offer some challenges (e.g. “what comes next?”, “I can never remember the second ‘S’”). More importantly, in this context, they put control of the encounter firmly in the hands (or mouths) of the facilitator. In an exploration of this published in this journal last year ( Norris and Bullock, 2017), the given mnemonic has 7 speech acts, only one of which is initiated by the learner. The group is not allowed into the conversation until Step 4, when they are invited to share perceptions of the event and this step is intended to encourage “.. honest reflective and constructive opinions ..” thereby building team membership and active listening. This seems unlikely to us: the model does not seem designed to encourage lively engagement but more likely, given the power imbalances, is structured to privilege the direction provided by the facilitator as they work through the mnemonic.

When we devised the learning conversation in 2007, a model (without mnemonic) was offered in the hope that people would have something to hang on to in order to replace the rigidity of the Pendleton model that had emerged in life support training. In providing a loose structure (or number of elements), we were aware of the risk associated with having a facilitator led agenda to explore. The Generic Instructor Course Virtual Learning Environment topic for Feedback alerted facilitators and learners to this risk when we wrote:

*“one of the problems .. is that you are thinking so hard about your opening question that you fail to focus on the candidate and their needs”* (GIC LMS [accessed 1 ^st^ February 2018])

In recognition of this challenge, we suggest “active listening” which creates an orientation within which the facilitator suppresses the urge to talk and instead gives room and time to explore the perspectives that are uppermost in the minds of the learners. The inevitable key component of this is the use of silence, and we are drawn to an example of this offered by an emergency department (ED) doctor and Advanced Paediatric Life Support (APLS) instructor in her online presentation at St Emlyn’s. In this paper, May depicts silence as “Wait time” and describes it thus:

*.. it occurs in two phases. Wait time 1 occurs after the question is posed (usually by the facilitator). Wait time 2 occurs after the respondent has stopped speaking and is the time between the end of the answer and the next thing the facilitator says.”*

Having space, not simply to think but even more significantly to verbalise, allows learners to go through a process in which vague thoughts become concrete, memorable (to them and others) and useful. One of the few acronyms that works for us relates to this: WAIT: Why Am I Talking? (DuckDuckGo, passim). This can act as a useful, if somewhat tongue in cheek, reminder of the importance of a learner-driven conversation.

We acknowledge that the learning conversation is a challenge and that many facilitators are caught in the bind of wanting structure while recognising that structure can lead them towards dominating the conversation. In recognition of this we have found that facilitators have responded well to a simple model which has just two strands, the second of which is often redundant when we genuinely provide space for the learners to initiate and collaboratively explore issues that have emerged from their practice.

**Figure 1.**
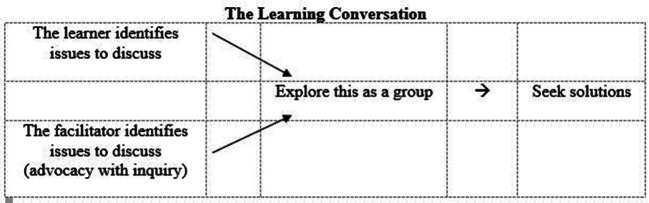
a model for the learning conversation

More often than not, learners, particularly those with more experience to draw on, identify the area that the facilitator would also want to raise. There are possible barriers to this, however, and among these is a resistance to exposing self to critical analysis by virtue of previous bad experience. Advocacy with inquiry, if well presented, can “give permission” to learners to open up their performance to supportive scrutiny. It is our view that lack of insight is not as widespread as is often claimed and it frequently masks an inability to guess what is going on in the mind of the facilitator.

## Maximising the Learning

**Figure 2.**
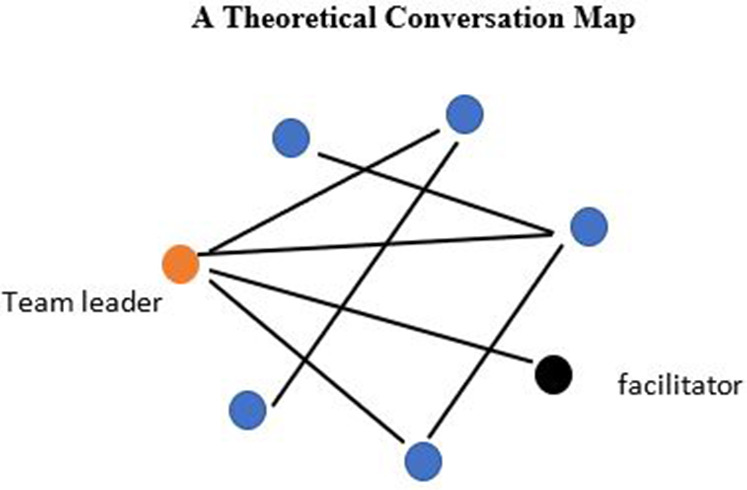
a conversation map for an effective learning conversation

Group discussion is key to the success of the learning conversation, providing appropriate support to the team leader, with evidence suggesting that they hear commentary better from peers in a process described as social proximity or cognitive congruence ( Bennett *et al*., 2015). Studies have shown that learners rate the quality of peer feedback as more useful than tutor feedback (for example English *et al.,* 2005). In fact we are describing a conversation which *includes* feedback, but is actually more than that, as often the meaning making occurs *during* the conversation and at its best is characterised by a flow which is illustrated in figure 2.

Group reflection is not only useful for the prime recipient of the learning conversation: it also leads to shared learning, allowing the whole group to make links with their own practice on the day or from previous relevant experiences. In addition it helps peers move closer to an understanding of what constitutes good practice as they evaluate and therefore calibrate where the benchmarks lie (Carless and Boud, 2018). Sometimes learners need a nudge towards identifying solutions as indicated in the model in figure 1, when they risk talking round the issues. This is a further opportunity for sharing, exploring and making suggestions rather than a time for facilitator-imposed solutions. It is our belief that students are much more likely, in the longer term, to act on solutions in which they have a personal investment.

As has already been identified, facilitators are encouraged to raise a significant issue *if it has not already emerged*, when, for whatever reason, the learner lacks the capacity or willingness to bring it up themselves. At the heart of the intervention is the notion of *advocacy with inquiry* ( Argyris, Putnam and Mclain Smith, 1985), described as a process designed to explore behaviour in complex settings and to examine barriers to more effective performance. Traditional models of feedback have often separated these two elements and encouraged:

• a lot of advocacy, where the person giving feedback sets the agenda and does most of the talking, or
• a lot of inquiry, where learners are invited to “guess what is going on in the head” of the person giving feedback

The learning conversation in contrast, is designed to integrate these and to encourage a more reflective orientation towards experience driven by the learners.

We are cognisant of the fact that it is challenging to be on the receiving end of A/I. This is because the advocacy may identify perspectives on behaviour that may seem critical. When used effectively, however, A/I strives not to privilege the voice of the facilitator. In contrast, the learner is invited to share their “frame” (their schema for the event) which might well be illuminating and surprising both to them and to the facilitator. A sample of what we have in mind might be:

*“When the patient went into PEA I noticed that you were looking at the monitor for about 10 seconds, and I wondered if you were unsure about the rhythm. When you looked at the rhythm, what went through your mind?”*

In this example, the facilitator makes a data based observation (the delay), and speculates as to the cause, before asking for clarification of the thought process. So, even though the facilitator is initiating this strand of the conversation, the focus is on the candidate’s thought process.

The time allocated to feedback on some of the life support courses is extremely brief in comparison to good practice in simulation scenarios, for example, and the learning conversation was designed with this in mind. At its best it allows the learner to instantly identify a concern, issue, high point, or challenge and to unpick this with the support of their group. There is also space for the facilitator to bring up a key issue either of technical or non-technical origin and again encourage exploration of that as a group. We consider that it is essential that the environment is one in which puzzles can be explored and is safe, and we have found that the best way for this to occur is to minimise hierarchy by empathy, honesty and a genuine interest in the perspective of others. What this means in practice is challenging ourselves to be silent, but vigilant and facilitate a group discussion around issues relevant to the learners.

## Take Home Messages

• students learn best when they generate the conversation and thinking themselves
• facilitators’ most important (and challenging) skill is the use of silence
• advocacy with inquiry can be a helpful way of raising issues that learners have not surfaced
• the learning conversation is an effective tool when debrief is time restricted

## Notes On Contributors

Dr Mike Davis is a freelance consultant in continuing medical education with extensive experience in a variety of course provision, both nationally and internationally. He is particularly interested in the contribution of adult education theory and group dynamics to effective CME.

Dr Kate Denning is a freelance consultant in continuing medical education who, with Dr Mike Davis, devised the learning conversation to improve feedback on life support courses. She has supported numerous communities in the transition to this form of feedback both in the UK and internationally.

## Declarations

Kate Denning is lead educator with the Advanced Life Support GroupMike Davis is past lead educator with the Advanced Life Support Group
