# Supplementary material for: Listening through the learning conversation: a thought provoking intervention
Source: MedEdPublish (2016). 2018 Sep 6;7:199. [Version 1] doi: 10.15694/mep.2018.0000199.1 (PMC10701807; doi:10.15694/mep.2018.0000199.1)
Supplement: Peer review history

# Reviewer report: Reviewer response for version 1

**DOI:** 10.21956/mep.19663.r29352

**Published:** 2018-09-07

**Authors:** Richard Hays

**Affiliations:** 1 James Cook University

This review has been migrated. The reviewer awarded 4 stars out of 5 Thank you for the invitation to review this interesting paper. I think it is a timely contribution to a topical debate about how much and what kind of feedback to provide in what time frame. Complaints from learners about insufficient feedback are loud and persistent, yet more and more feedback is provided. Learners can now receive detailed data on quartile performance, comparision with mean cohort scores and references to readings that (eventually) provide the right answers, often generated automatically by assessment software and delivered to personal inboxes. However, this paper reminds us that the MEANING to the learner may be much more important than the increasing amount of factual feedback. The model of learners discussing the data, seeking the views of their peers and reaching a shared understanding is appealing to educators because it places the feedback more in the hands of learners, who are the main beneficiaries. The discussions generated are likely to be more valuable than reading the facts, which afterall are mostly only numbers linked to grades. It is more important to understand where strengths and weaknesses exist, including the nuances of what makes the 'right' answer better than others. Group feedback, facilitated by an experienced educator in a similar role to a PBL tutor, may well be an effective, although more expensive, way of maximising learning from assessment performance. The tutor manages the conversations, stepping in to encourage reflection on cognition and judgement. The model seems adaptable to any form of assessmnet method. At this stage the idea has a reasonable theoretical basis, but needs research to test the impact on future performance.

Reviewer Expertise:

NA

No decision status is available

# Reviewer report: Reviewer response for version 1

**DOI:** 10.21956/mep.19663.r29353

**Published:** 2018-09-06

**Authors:** Trevor Gibbs

**Affiliations:** 1 AMEE

This review has been migrated. The reviewer awarded 5 stars out of 5 I too really enjoyed reading this paper, simply because I learned a lot about communication and it made me think about the standard models of feedback and how they can be improved. As GP by background, I always considered myself quite good at communication skills and often was involved in teaching in that area. As an older GP, I learned mainly through Pendleton rules, especially when giving feedback to my trainees, but this paper made me question whether I used these standard approaches appropriately. This paper is very well written, well-structured , and with an easy flow to it. I would agree that there area lot of new referenced works out there that raise the bar as regards feedback, but the references used are appropriate for what the author is saying and hoping to show.I would suggest that this paper is relevant for all those involved in teaching and learning in the health professions as well as those involved in the teaching of communication.

Reviewer Expertise:

NA

No decision status is available

# Reviewer report: Reviewer response for version 1

**DOI:** 10.21956/mep.19663.r29351

**Published:** 2018-09-06

**Authors:** Subha Ramani

**Affiliations:** 1 Harvard Medical School, Brigham and Women's Hospital

This review has been migrated. The reviewer awarded 4 stars out of 5 I enjoyed reading this article for a number of reasons. First, feedback is one of the most challenging interpersonal interactions between teachers and learners. Second, it has been the subject of my own recent research. Third, I agree with the authors that while feedback is integral to professional development, it needs to move away from the senior pundit-junior apprentice approach.For this reason, I find this concept of the learning conversation very timely and appropriate to take feedback to the next level. I also agree that formulae and mnemonics can lead to an artificial and stilted conversation where feedback providers are distracted by what comes next. Based on the definition of feedback by Boud and Molloy, impact on the recipient should be the focus rather than 'how to'. For this reason, learners initiated feedback conversations might enhance the credibility of the content and raise acceptability of the data. The authors also bring a new dimension into the feedback conversation, that of group reflection.The paper is well written, anchored by relevant theory.However, the references are sparse. There is a large body of recent research on feedback that focuses on psychosocial theories, relationships between teachers and learners, newer models that emphasize relationships / alliance and place the learner at the heart of the conversation. I do believe these should have been included as they are landmark articles in this field.All health professions educators and learners, regardless of research interests, will find this article useful, but I will still recommend reading other published articles to gather the whole picture.

Reviewer Expertise:

NA

No decision status is available
